# Robust sensing suite for measuring temporal dynamics of surface temperature in sewers

**DOI:** 10.1038/s41598-018-34121-3

**Published:** 2018-10-30

**Authors:** Karthick Thiyagarajan, Sarath Kodagoda, Ravindra Ranasinghe, Dammika Vitanage, Gino Iori

**Affiliations:** 1 Centre for Autonomous Systems, University of Technology Sydney, Sydney, NSW 2007 Australia; 2 Sydney Water Corporation, Parramatta, Sydney, NSW 2150 Australia

## Abstract

Sewerage systems are paramount underground infrastructure assets for any nation. In most cities, they are old and have been exposed to significant microbial induced corrosion. It is a serious global problem as they pose threats to public health and economic repercussions to water utilities. For managing sewer assets efficaciously, it is vital to predict the rate of corrosion. Predictive models of sewer corrosion incorporate concrete surface temperature measurements as an observation. However, currently, it has not been fully utilized due to unavailability of a proven sensor. This study reports the feasibility of infrared radiometer for measuring the surface temperature dynamics in the aggressive sewer conditions. The infrared sensor was comprehensively evaluated in the laboratory at different environmental conditions. Then, the sensor suite was deployed in a Sydney based sewer for three months to perform continuous measurements of surface temperature variations. The field study revealed the suitability of the developed sensor suite for non-contact surface temperature measurements in hostile sewer conditions. Further, the accuracy of the sensor measurements was improved by calibrating the sensor with emissivity coefficient of the sewer concrete. Overall, this study will ameliorate the present sewer corrosion monitoring capabilities by providing new data to models predicting sewer corrosion.

## Introduction

Urban sewerage systems transporting domestic and industrial wastewater through underground pipelines have been widely regarded as an imperative infrastructure asset of our society mainly for the reason that they conserve our civic communities from the menace of sewage-borne diseases, abhorrent odors and unhygienic conditions^1^. Majority of these sewers are built using the concrete material and microbial corrosion in these types of sewers has historically been a significant problem for the wastewater utilities around the globe. In fact, the first occurrence of sewer corrosion was noticed in the late 19^th^ century in USA^2^. However, it was only in the 1940s, the methodological studies conducted in Australia and USA entrenched the biological nature of concrete corrosion^3–5^. Then in the 1980s, a radical increase of concrete corrosion was observed in the sewers of USA^6^ and Europe^7^. Until that time, sewer corrosion was not regarded as a major problem. Increases in sewage temperature due to urbanization factors such as the discharge of household detergents containing sulfur and toxic metals from industries were related to the ascendancy of sewer corrosion in 1990s^8,9^. Currently, the value of sewer infrastructure assets in USA and Australia are estimated to be over $1 trillion and $100 billion respectively^10^. This is nearly equivalent to 7% of USA’s and 6% of Australia’s gross domestic product as of 2016. Failure to maintain such infrastructures will lead the utilities and governments to a range of economic repercussions. For example, the sewer assets are damaging at an estimated annual cost of $13.75 billion in USA^11^, $50 billion in Germany^12^ and $100 million in Australia^13^. So, the cost for restoration and maintenance of sewer assets are expected to increase as the failure of critical sewer pipes continues^14^.

In Australia, nearly 110,000 kilometers of sewer piping benefit various sectors of the community^15^. Under anaerobic conditions, the sulfate-reducing bacteria in those sewer pipes produces dissolved hydrogen sulfide (H_2_S) in wastewater^16^. Due to turbulent flow, the dissolved H_2_S present in wastewater is released to sewer atmosphere as gaseous H_2_S^17^, which is absorbed into the exposed concrete surface, notably on the crown of the sewer pipe. Due to the occurrence of biological oxidation, the H_2_S on the concrete surface is converted into sulphuric acid (H_2_SO_4_) by the micro-organisms that dwell on the moist surface of the concrete^14^. The generated biogenic H_2_SO_4_ penetrates into the pores of the concrete and starts to chemically react with the cement material and initiates corrosion^18^. Presently, the buried concrete sewer pipes are vulnerable to structural deteriorations mostly due to the physical and biological process known as Microbial Induced Corrosion^19^. In pursuance of managing the sewers effectively, the water utilities are committed to comprehend the elements that leverage corrosion.

One of the direct measures to address the sewer corrosion problem is to measure the amount of concrete corrosion itself. But, there is no reliable, robust and efficient technology in the state-of-the-art systems to measure this quantity throughout the network. The micro-organisms that are responsible for producing biogenic H_2_SO_4_ on the concrete surface could be a good proxy to measure corrosion. However, this involves the tedious task of identifying the liable bacterial species from other micro-organisms that reside on sewer pipes. The next level of proxies that could indirectly indicate the amount of concrete corrosion would be the factors that provide favorable living conditions to bacteria. In this sense, H_2_S levels in sewer air, optimal temperature and moist conditions of the sewer wall attributes to bacterial activity^13,20^. Presently, there is an industry-proven commercial system to monitor H_2_S levels in sewers^21,22^ and research is underway for monitoring moisture near the sewer concrete wall using fibre optic technology^23^. However, there are no state -of-the-art systems to measure surface temperature conditions in sewers. So, the unavailability of technology to quantify that proxy measure leads to choosing another level of proxy, which is the gas-phase temperature of the sewer air. However, in locations where the sewer pipe is above the ground level, the surface temperature near the crown can be intermittently distinctive to the temperature of the sewer atmosphere mainly due to the degree of moisture condensation that occurs within the concrete pore structure. Therefore, the temperature of the sewer air is not a representative measure to that of sewer wall temperature.

Recent studies have shown the feasibility of measuring different temperature variables in the sewer. For example, the gaseous temperature of sewer air was measured inside the corrosive sewer pipes in different cities of Australia^13,20^. Similarly, the effluent temperature and ambient temperature of the sewers were measured in two sewer manholes of the Kent city in England and thereby observed that on average effluent temperature is higher by 3.5 °C^24^. A considerable amount of research was performed using Distributed Temperature Sensing (DTS) technology, which utilizes fibre optic cables for measuring the wastewater temperatures in sewer networks^25,26^. The use of DTS technology has been demonstrated in the application of measuring temperature gradients at different positions of the sewer pipe by placing the fibre optic cable near soffit (top), wastewater level (floating) and invert (bottom)^27^. Although researchers have focused on measuring different temperature variables in the sewer, there have been no reports in the scientific literatures about the measurement of concrete surface temperature in sewers. As the sewer corrosion is dependent on the surface temperature variable, the water utilities are very keen on sensor technology for measuring the surface temperature at the crown of sewer pipes.

In recent years, researchers have attained several landmarks on sewer corrosion modelling using ambient temperature, relative humidity (RH) and H_2_S levels^20^. Despite the fact that such works are progressing towards conceivable results for corrosion prediction, there is still a large proportion of uncertainty cohered with the model prediction. Hence, it is significant to provide new data regarding the temperature of the concrete surface as it favors the bacterial activity. In this context, the objective of this collaborative research between the University of Technology Sydney and Sydney Water is to tackle the aforementioned problem by developing a surface temperature monitoring suite that can operate under the aggressive environmental conditions of the sewers. The Sydney Water is a New South Wales Government-owned statutory corporation and Australia’s largest water utility that delivers sustainable drinking water and wastewater services to over 4 million people. Since the sewers are classified as Zone-2 hazardous area in Australia^28^, there are multifarious requirements to be considered in developing a sensor suite as there are no commercial sensor systems proven to be sewer deployable and comply with the requirements specified by the sewer operators. In the light of the preceding discussion, the sensor suite should possess properties such as low maintenance and convenient access to the monitoring data, easily deployable and removable, non-destructive measurements on the concrete surface at regular frequencies, integrated multi-sensor package to accommodate different sensors and sustain in harsh sewer conditions. In order to accomplish this, several laboratory studies were conducted to recognize radiometry based surface temperature measurements as a potential option to address the key challenges emerging from the sewer environments^28–30^. Before installing the actual sensor suite, a pilot study was conducted in the municipal sewer of the Sydney city in Australia to examine the ruggedness of a stainless steel 316L grade sensor enclosure having wiper mechanism^28^. As an outcome of that study, the design of the sensor enclosure was re-engineered based on the feedback from sewer operators. Further, it was observed that the enclosure optics tends to fog up in the sewer humidity conditions. To mitigate the effects of sewer humidity during the sensor measurements, a germanium made optical window having 8–14 *μm* wavelength was chosen. Several laboratory experiments were conducted in the humidity chamber to embrace antifog glazed optical window after evaluating other approaches including electrical heating of the optical surface for thwarting surface fogging.

This paper reports a comprehensive evaluation of an infrared radiometer (IRR) sensor for real-time continuous measurement of surface temperature in sewers. In the laboratory study, the performance of optical window, the effects of incident angle, limit of detection, distance, lighting condition, reproducibility, humidity conditions, varying surface temperature conditions were investigated. Thereafter, the sensor was deployed in the sewers for evaluating the long-term sensing performance and endurance of the sensor package. The measurements from a non-contact type IRR sensor was examined with the measurements from a contact-type thermistor sensor to furnish a scientific evidence for supporting the application of non-contact surface temperature sensing in the sewer. In addition, the quantifications of ambient temperature *in*-*situ* and *ex*-*situ* of the sewer pipe were compared and analyzed with the measurements resulting from the IRR sensor. After successfully completing the field trial campaign, the sensor suite was brought to the laboratory for post-deployment investigations. Further, the measurements from IRR sensor data was improved by performing emissivity based temperature correction. To the best of our knowledge, this is the first time that a paper reports the feasibility of non-contact monitoring of surface temperature in the aggressive sewer environment with a motive of augmenting the present development of corrosion modeling and predictive analytics.

## Results

Following section elucidates the experimental results carried out in the laboratory and field conditions using the IRR sensor.

### Assessment of IRR sensor’s optical window in varied humidity conditions

**Figure 1:**
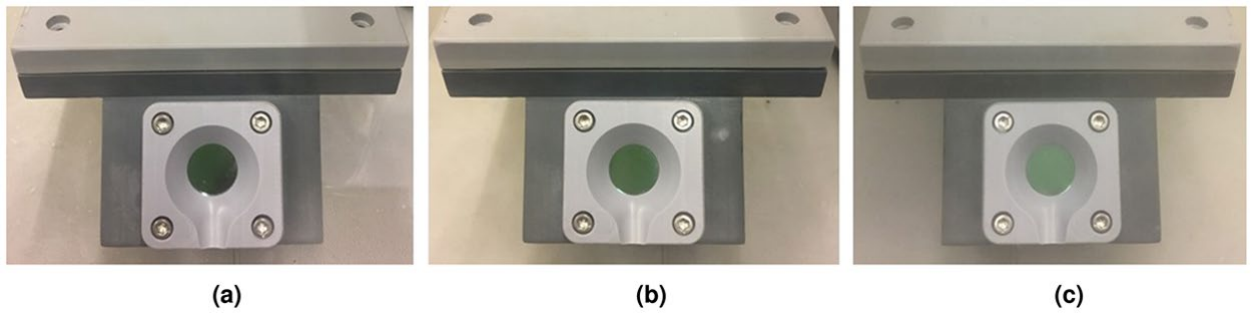
Experimental evaluation of the optical window exposed to different relative humidity conditions inside the humidity chamber. (**a**) 80% RH (**b**) 90% RH (**c**) 100% RH.

This test demonstrates the laboratory assessment results of the IRR sensor’s optical window after exposing to different humidity conditions inside a humidity chamber. Two approaches were shortlisted to mitigate the effects of fogging on the optical window surface. The first approach needs micro-controller based electrical heating of the optical window surface above ambient temperature so that it prevents the surface fogging. Although this approach yielded desired outcomes, it needs an additional power supply to keep the electrical circuitry active all the time. The second approach uses antifog coating material on the surface of the germanium made optical window and thereby avoids the need of additional power source to prevent surface fogging. This approach was evaluated inside a humidity chamber in different humidity conditions such as 80% RH, 90% RH and 100% RH. Figure 1 presents the images of optical surface exposed to different humidity levels in the test chamber. The images appear to be gloomy because they were captured in high humidity conditions inside the chamber. The careful visual inspection revealed the surface of the germanium made anti-fog coated optical window led to no surface fogging. Therefore, this antifog coated optical window was used in developing the sensor suite.

### The effects of incident angle on the IRR sensor performance and their limit of detection

**Figure 2:**
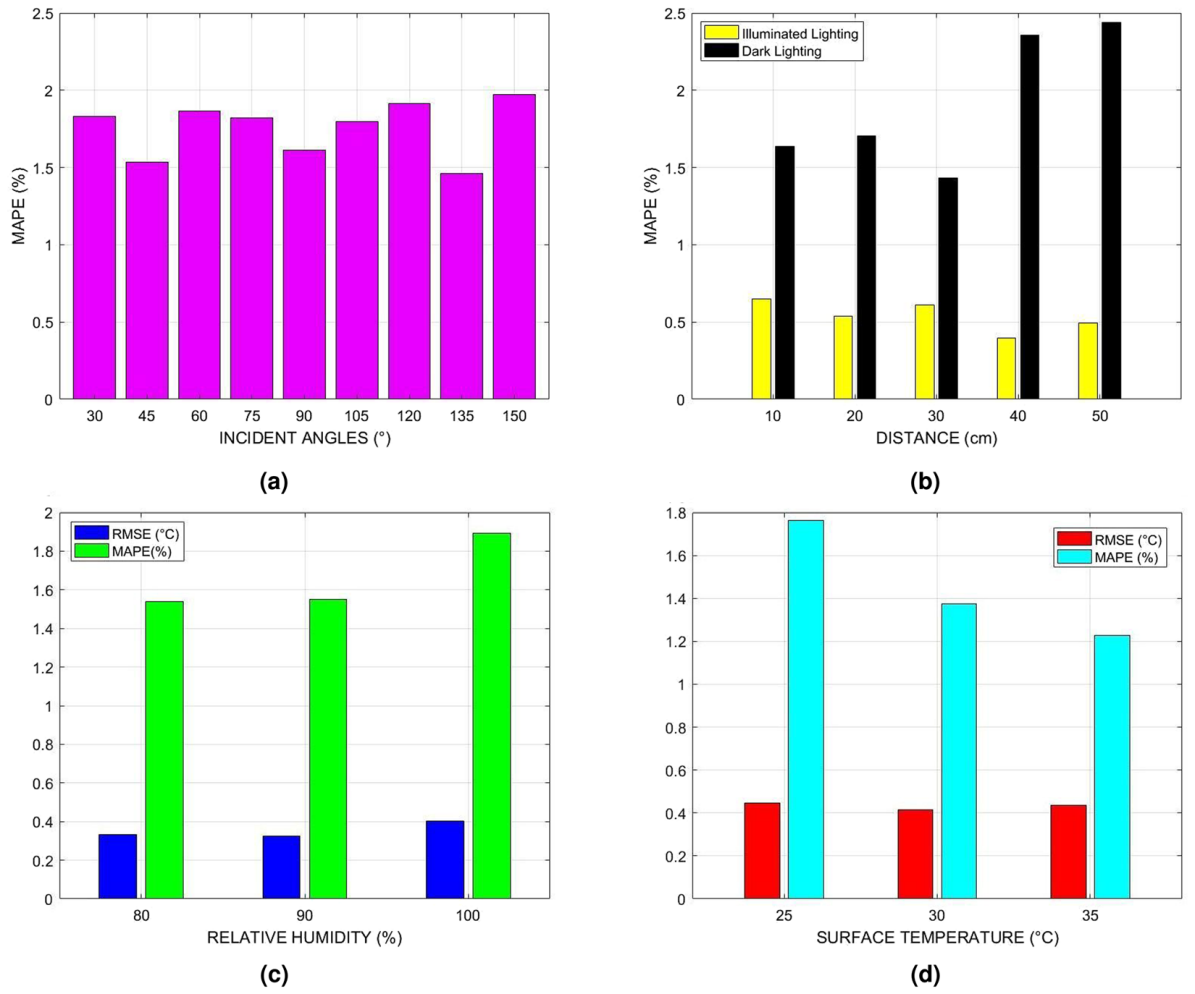
Statistical calculation of MAPE and RMSE for evaluating the IRR sensor under different conditions. (**a**) Performance efficacy of IRR sensor by positioning it at different incident angles from the surface of interest. (**b**) Statistical comparative analysis of IRR sensor measurements obtained in illuminated and dark ambient conditions by positioning the sensor in different distances. (**c**) Computed measures of RMSE and MAPE illustrating the IRR sensor performance under different humidity conditions. (**d**) IRR sensor performance with varying surface temperature.

This test evaluates the sensing performance of the IRR sensor by placing it at different incident angles from the surface of interest in dark lighting condition similar to confined sewers. Two surface temperature sensors, i.e. one IRR sensor and one epoxy coated thermistor sensor were used in this study. The measurement of the thermistor sensor was used as a reference for all the investigations carried out. The performance was evaluated by computing Mean absolute percentage error (MAPE). Figure 2a presents the statistically calculated proportions of MAPE for different incident angles, where it can be observed that the variations were relatively small (≤0.5%) with no obvious pattern. The relative difference between the sensor and reference measure being less than 2.5% is generally regarded as reliable sensing^21,22^. In this test, the MAPE for the IRR sensor measurements is less than 2.5% implying the effects of the incident angle between 30° and 150° is reliable and has an imperceptible impact on the IRR sensor performance. However, the half angle field of view of the IRR sensor which is 22° needs to be considered while mounting the sensor.

### Distance, lighting condition and reproducibility

In this test, the sensing performance of the IRR sensor is studied in the laboratory by positioning the sensor at different distances from the surface of interest under varying lighting condition. This test was conducted mainly to recognize the pertinent distance between the IRR sensor and target surface at the time of installing the sensor suite inside the sewer pipe. MAPE was used as a statistical metric for computing the relative percentage difference between the measurements of IRR sensor and the reference measure. Figure 2b presents the computed MAPE (%) for the measurements obtained at different distance and lighting condition, where it can be observed that the percentage of MAPE is higher for the dark lighting conditions than the illuminated lighting condition for all the distance between 10 cm and 50 cm. However, the effects of distance in a particular lighting condition had a negligible impact on the performance of the IRR sensor. MAPE was utilized to study the reproducibility of the IRR sensor data. From the Fig. 2a,b, it can be observed that the MAPE for the IRR sensor performance under different incident angle, distance and lighting conditions is smaller than 2.5%. This implies that the IRR sensor has good reproducibility similar to the sensor monitoring methane in sewers^21,22^.

### Performance of IRR sensor in higher humidity conditions

In general, the RH conditions of the sewer are over 80%^20^. In this test, the performance of the IRR sensor was evaluated in different RH levels (80%, 90% and 100%) at the laboratory. The root-mean-square error (RMSE) and MAPE were used as performance measures to evaluate the IRR sensor performance. Figure 2c presents the computed RMSE and MAPE by using the IRR sensor readings and the reference readings. It can be observed from the Fig. 2c that the RMSE for the range of 80-100% RH was less than 0.5 °C and shows no symmetric trend for the different humidity levels. This indicates that the IRR sensor can be used with no further calibration as the IRR sensor is factory calibrated for operating in 0–100% RH conditions. Similar to the aforementioned laboratory experimental results, this test results present MAPE less than 2.5% for the conditions of 80–100% RH (Fig. 2c). These results clearly demonstrate the performance of IRR sensor is not affected by the high humidity conditions and provides credible measurements.

### IRR sensor performance with varying surface temperatures

In this laboratory experimentation, we evaluated the IRR sensor by measuring the increased surface temperature inside the humidity chamber having 100% RH. The increased surface temperature levels were 25 °C, 30 °C and 35 °C. Figure 2d presents the computed RMSE and MAPE for comparing the IRR sensor performance at different temperature levels. It can be noted that the RMSE for all the three temperature levels were approximately 0.4 °C. However, the MAPE showed a decreasing trend from 25 °C to 35 °C. This is due to the reason that the proportions between the reference measure and IRR sensor measure decreases while the absolute error between them remains around 0.4 °C. The outcomes of the results were reasonable and indicate that the IRR sensor can acquire effective measurements under increasing surface temperature levels.

### Field deployment of a sensor suite for real-time continuous measurements in a confined sewer

**Figure 3:**
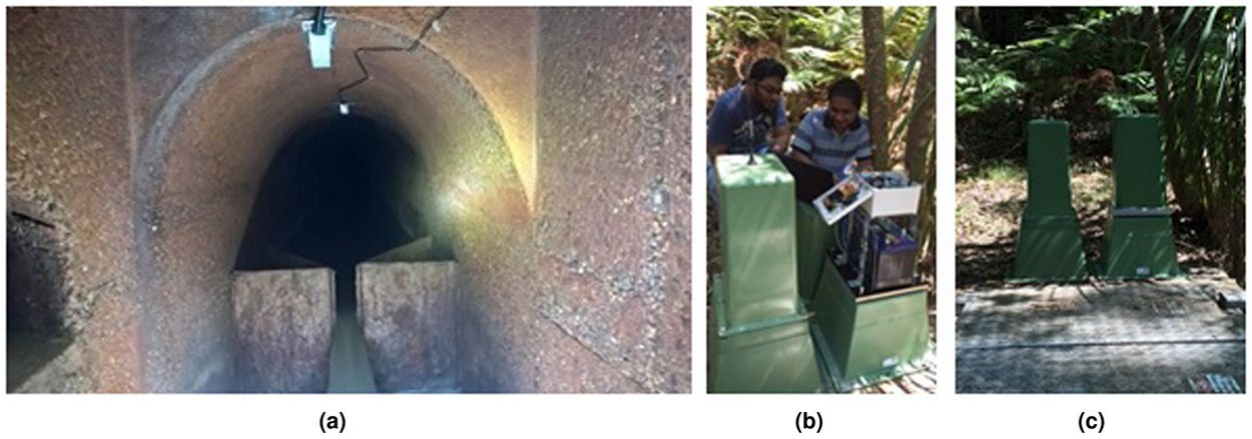
Field deployment of a sensor suite at a sewer site for real-time measurements of temperature variables. (**a**) Installations of the sensing unit at the crown of the sewer pipe for monitoring surface temperature variations. (**b**) Construction of the monitoring unit outside the sewer pipe. (**c**) Housing the monitoring unit using an electrical pillar box having vented air supply.

An advanced sensor suite comprising sensing and monitoring unit was developed in the laboratory and was introduced to the real sewer environment for evaluating the sensing performance and the durability of the packaged sensors. Based on the recommendations of the sewer operators from the Sydney Water, a sewer site at the Thornleigh area in the municipality of Sydney, Australia, was chosen for deploying the sensor unit. This site was at a remote location, where there is no access to mains power and therefore, a long-term battery powered operation was a requisite. The sensing unit was installed near the crown of the sewer pipe for measuring the surface temperature variations (Fig. 3a) whereas the monitoring unit was constructed outside the sewer pipe for accessing the data (Fig. 3b,c). This field testing campaign was conducted for a period of about three months. Although this study emphasizes on measuring surface temperature variable in the sewer system, other temperature variables that may impact the surface temperature like the ambient temperature inside sewer pipe and ambient temperature of the field site (outside sewer pipe) were also measured during the field trial campaign.

The sensing unit comprises two temperature sensors namely an IRR sensor and an epoxy coated thermistor sensor. Both the sensors measure surface temperature variations on the exposed concrete surface. The IRR sensor works on the principle of Stefan-Boltzmann Law^28^. The sensor converts the thermal radiations from the surface of interest into an electrical signal, which is used to compute surface temperature without contacting the surface. The thermistor sensor was used as a reference measure and it is a contact type sensor, which performs measurements by touching the surface of the sewer concrete wall. This sensor is made up of a semiconductor material that varies the resistance based on the sensing temperature. Both the sensors were housed in a tailor-made enclosure, which is made up of polyvinyl chloride (PVC) material. This enclosure allows the tip of the thermistor to be in contact with the concrete surface while the body of the thermistor is insulated by the enclosure. Besides the surface temperature sensors, another two thermistor sensors were used to measure the temperature of the sewer air and ambient temperature of the field site. After each measurement, the sensor data was transmitted to the monitoring unit by using a 20 meters long cable. It was brought to our knowledge during the discussions with the sewer operators that the sensor cables transmitting signals need to be animal proofed mainly due to the reason that the vermin in sewers often nibble the cable sheathing. For this reason, the signal transmitting cables were placed inside an electrical conduit from the sensing unit to the monitoring unit.

In the monitoring unit, the incoming analog signals from the temperature sensors are converted into a digital signal by the signal processing unit. Then, the processed digital signal is sent to a logging instrument, where the data is stored after each measurement with the respective time-stamp. The sensor system is set to perform measurements at an interval of one hour in hour boundaries. From the data logger, the sensor measurements data can be accessed and downloaded in the form of a comma separated values file (.csv). Due to the intrinsic safety concerns of the sewer, a DC battery was used to power the sensor system placed in the outside monitoring unit. The entire monitoring unit was housed inside an electrical pillar box with vented air supply at the field site (Fig. 3c).

### Real-time sensor data showing temporal dynamics of surface temperature measures from the sewer pipe

**Figure 4:**
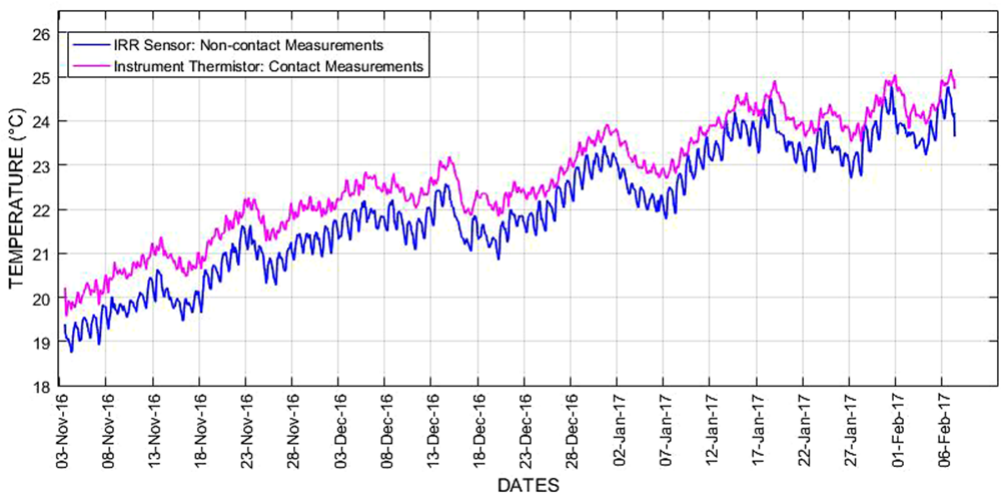
Surface temperature profiles obtained from IRR sensor and reference instrument thermistor measurements.

The surface temperature data obtained from the sewer monitoring campaign using IRR sensor is shown in Fig. 4, where it can be noticed that there is no sharp variation of surface temperature measures between the days. The monthly average of surface temperature measurements for November 2016, December 2016 and January 2017 was 20.30 °C, 21.89 °C and 23.25 °C respectively. For the first week of February 2017, the average surface temperature was 23.88 °C. Overall, the average surface temperature measure obtained from the IRR sensor during the field trial was 22.22 °C. Figure 4 also presents the surface temperature data acquired with the reference instrument thermistor sensor along with the IRR sensor measurements for profile comparisons. From both the sensors, the dynamics of the surface temperature profiles were captured reasonably and displayed a similar pattern throughout the field trial. In general, the diurnal pattern tends to vary approximately less than ±1 °C. The mean relative difference between the measurements of two sensors was 0.67 °C. Overall, the surface temperature data from both the sensors showed an increasing trend from the month of November 2016 to February 2017 over the summer season of the Sydney city.

### Comparative analysis of IRR sensor data with ambient and field temperature data

**Figure 5:**
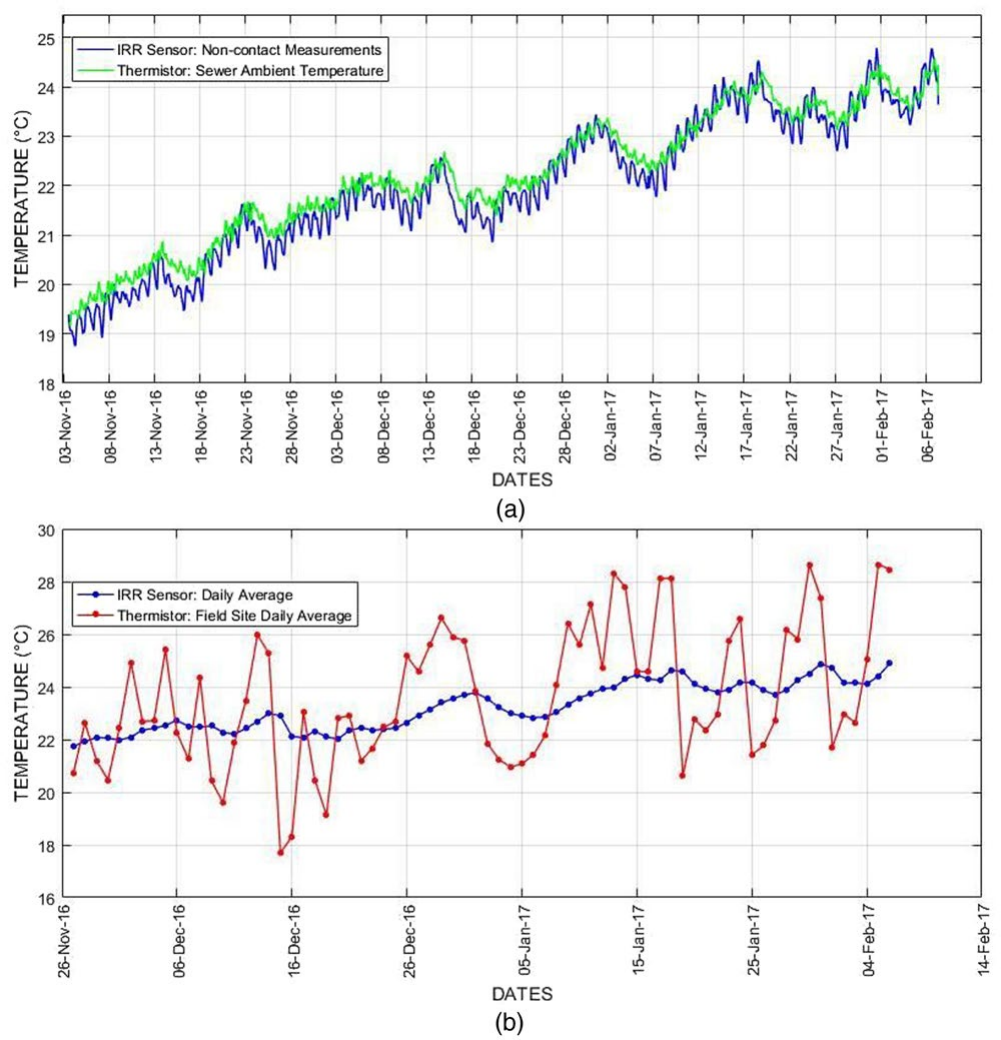
Comparison between the surface temperature profile and the ambient temperature profiles. (**a**) Comparison of surface temperature profile from IRR sensor with the sewer ambient temperature profile from the thermistor sensor. (**b**) Comparison of daily average surface temperature profile from IRR sensor with the daily average of ambient temperature outside the sewer pipe.

Figure 5a shows the profile of ambient temperature inside sewers measured by the thermistor sensor and the surface temperature profile acquired using IRR sensor. The sewer air temperature and the surface temperature tends to follow the similar pattern. However, there is a slight difference between the two variables due to the reason that the surface takes time to be in equilibrium with the atmospheric temperature. Figure 5b presents the daily average values of surface temperature measurements from IRR sensor and the ambient temperature of the field site. A thermistor sensor was installed on 26^th^ November 2016 to measure the ambient temperature outside the sewer pipe. In Fig. 5b, it can be recognized that the average daily surface temperature remains to be increasing as long as the ambient temperature outside sewers (field location) increases. In contrast, the surface temperature pattern tends to decrease when the ambient temperature of the field location decreases. This phenomenon implies that the surface temperature of the sewer tends to behave as the dynamics of the sewer air temperature, which synchronizes with the ambient temperature outside temperature of the sewers.

### Improving surface temperature measurements from IRR sensor by emissivity coefficient correction

**Figure 6:**
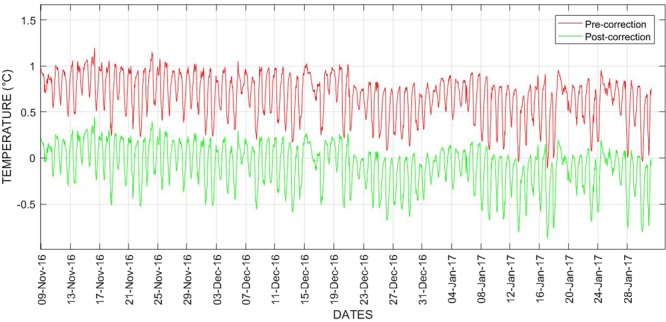
Improving surface temperature measurements of the IRR sensor between November 2016 and January 2017.

The IRR sensor used in developing the sensor suite was calibrated to provide surface temperature measurements for surfaces having emissivity value approximately 0.98. As the emissivity values can vary in different surfaces, there is a need to determine the emissivity value of the surface of interest for providing accurate measurements. In this regard, a method of field calibration was introduced to determine emissivity value of the sewer concrete surface during the Sydney summer period. These emissivity coefficients of the sewer concrete surface were used to enhance the temperature measurements. The pre-correction data is determined by the relative difference between the reference instrument thermistor and IRR sensor readings. After determining the emissivity coefficient, the surface temperature is improved by correcting the measured readings. This data is termed as post-correction data. For the surface temperature correction analysis, surface temperature data of about 5 days between 03^rd^ November 2016 and 08^th^ November 2016 were taken as a sample dataset for determining emissivity coefficient. Surface temperature measurements were corrected by using the data from 09^th^ November 2016 to 31^st^ January 2017. Figure 6 shows the plots of error differences between the pre and post correction IRR sensor data. The RMSE was used as a performance metric for evaluating the surface temperature correction performance. The RMSE of the pre-correction data and post-correction data is 0.72 °C and 0.25 °C respectively. From the performance evaluation, it can be observed that the surface temperature measurements can be improved when the emissivity coefficient is determined.

### Post-deployment validations of the IRR sensor after long exposure to harsh environmental sewer conditions

In this experimentation, the sensing performance of the IRR sensor was evaluated with the sewer exposed germanium optical window and with the new germanium optical window. The reference instrument thermistor sensor was used as a benchmark against the IRR sensor measurements and thereby MAPE was computed between the two sensor measurements. The MAPE for the IRR sensor measures using the new optics was 1.41% and for the IRR sensor measures using the sewer exposed optics was 2.21%. Despite the exposure of the IRR sensor to the aggressive sewer conditions, the MAPE of the IRR sensor is less than 2.5%, which was the case even in the laboratory experiments under dark lighting conditions prior to the field deployment. Therefore, this evaluation demonstrates the IRR sensor is in good condition with reliable sensing measures.

## Discussion

Although there are several sensors commercially available to measure surface temperature variations, their suitability for in-sewer environment has not been investigated till date. In this study, we used two different types of temperature sensors: contact and non-contact. The IRR sensor with anti-fog coated germanium optical window was used for non-contact type measurements. There was a slight visual degradation observed on the anti-fog coated optical window after 96 days of exposure to the aggressive conditions of the sewer. The degradations were in the initial stages around the edges of the optics and the central part remains unaffected. As a part of preventive maintenance, it is recommended to replace the lens once in three months for accurate measurements. The used optical window is having wavelength of 8–14 *μ*m. So, it will minimize the interference from atmospheric absorption or the effects of water vapour below 8 *μ*m and above 14 *μ*m. Also, the possible effects of gaseous compounds like NH_3_, H_2_S and volatile organic compounds like CH_4_ during sensor measurements can be minimal if the absorption bands are outside wavelength of 8–14 *μ*m. From the field experimentation, it can be said that the gaseous compounds having wavelength between 8 *μ*m and 14 *μ*m has less effects on sensor measurements. On the other hand, the contact-type sensing using thermistors can be a cheaper option to measure surface temperature in sewers. The sensor enclosure was visually inspected after the field trial campaign. No visual damages were apparent on the sensor enclosure other than a slight decoloration of the material. Both the sensors survived the three months period of field trials and the measurements generated by these sensors were reasonable.

The sewer site used in this study has no access to any electrical mains. In order to operate the sensor system, other means of supplying power to the monitoring unit was necessary. The option of using solar panels was not preferred by the sewer operators mainly because of their past experiences, where solar panels were damaged or removed by some external party. So, a DC battery was used to power the sensor system. Due to high power consumption of the sensor system, the DC battery was replaced with the recharged ones every week throughout the field testing. To mitigate this issue, presently we are exploring the options of using micro-controller based data logging instrument.

It can be concluded that all the sensors performed reasonably well and the measurements generated by them were legitimate. However, there were few anomalies found during the trials. The surface temperature data from the IRR sensor contained 0.35% of anomalies and whereas the data from the reference instrument thermistor contained 0.3%. The sewer air temperature variable measured from the thermistor sensor comprise of 0.3% of anomalies. All the anomalies were removed manually for computation and analysis. Further, the IRR sensor data was improved by correcting the surface temperature measures through the determination of emissivity coefficient. It can be further improved by determining the emissivity coefficient at regular intervals rather than the seasonal periods. In this study, we have only shown an approach to improve the surface temperature measure. Further study on determining the properties that determine emissivity will open the possibilities of improving the surface temperature automatically.

The conventional method of sewer corrosion modelling practice incorporate sewer air temperature as an input variable for estimating concrete corrosion throughout the sewer network. However, in the geographical areas where the concrete sewer pipes are located above the ground level, the surface temperatures of the concrete sewer pipes can be different to the sewer gas temperatures near the crown (headspace)^31^. This can affect the moisture condensation process that happens within the pores of the concrete sewer walls. Hence, the surface temperature of the concrete sewer can influence the condensation process and the corrosion activity. Due to unavailability of proven sensors in the state-of-the-art technologies to provide surface temperature measurements for the corrosion predicting models, the water utilities use surrogate measures like the sewer air temperature, which results in high uncertainties in prediction. Concrete corrosion levels in sewer pipe are estimated to be 70% higher when the surface temperature of the sewer pipe is colder by 1 °C relative to the sewer air temperature near the crown of the sewer pipe^32^. Therefore, the accuracy of surface temperature measurements lesser than 1 °C is vital in estimating the concrete corrosion in sewers. However, water utilities will evaluate the impact of air temperature versus the surface temperature data from the developed sensing system in their model for better corrosion prediction.

To the best of our knowledge, this paper is the first one to report the monitoring of surface temperature variations inside concrete sewers for the prolonged period of time through non-contact and contact type measurements, where both the sensors showed a similar trend. In contrast to the contact type measurements by the thermistor sensor, the IRR sensor facilitates non-contact measurements. This enables the sensor to measure the surface temperature variations without altering or having any physical contact with the exposed concrete sewer walls. This advantageous feature of the IRR sensor allows to monitor the surface temperature variations of the concrete surface without hampering the moisture condensation process that occurs on the pores of the concrete sewer walls, which has a significant impact on sewer corrosion. In addition, the degradations that can happen on the surface of the concrete sewer and the accumulation of algae or fungi on sewer walls will not affect the IRR sensor monitoring as it does not have physical contact with the concrete surface. In the case of thermistor sensor, degradation of the contact part of the concrete sewer surface may not provide the actual surface temperature measurements and may also hinder the moisture condensation. For the concrete sewer pipes having smaller diameter size, there are practical restrictions for human access in deploying the sensor system. In such pipes, sensors can only be installed in the either sides near the manholes. As the IRR sensor has demonstrated physical robustness to the hostile sewer conditions during the field testing, it can be mounted on a robotic or floating platform to perform non-contact measurements during sewer pipe traversing. Those measurements are vital for spatial estimation of surface temperature conditions of the sewer pipe and for sewer corrosion modelling.

In the field study, the sensor system was set to perform measurements in hourly intervals due to following reasons. Each measurement draw approximately 50 mA external power supply current. Increased frequency of measurements results in high power consumption and the battery life will be decreased. However, the sensor system can be programmed to take measurements at different intervals. In addition, temperature in sewers is a slowly moving quantity^24^.

In summary, this study has developed a robust sensor technology for monitoring temporal dynamics of the surface temperature inside the sewer pipes. From the field testing experiments, the study has revealed that the IRR sensor with an anti-fog coated germanium optical window can be used for non-contact surface temperature measurements in sewers. This sensor is more suited for deployments of about three months period continuously or can be used on moving platforms during human traversing. For long-term sensing operations using IRR sensor, it is recommended to replace or coat the optical window with anti-fog material after three months of use. The sensor enclosure demonstrated robustness under the aggressive environmental conditions of the sewer. We believe the real-time continuous measurements from the developed IRR sensor suite will provide information-rich data to the analytical models for better prediction of corrosion in sewers. The collaborators of this project will deploy the developed sensing suite in locations where there is less confidence in corrosion prediction^33^, in order to improve their predictive model. Hence, this manuscript only reports the viabilities of monitoring surface temperature variations and therefore, the manuscript has not combined with the models of sewer corrosion with the surface temperature measurements. Overall, this study can enhance the wastewater utilities present sewer corrosion monitoring capabilities.

## Methods

### Sensing Suite

Two surface temperature sensors, i.e. one non-contact type IRR sensor (Apogee SI-111, ICT International) and one contact type thermistor sensor (THERM-EP, ICT International) were chosen for both laboratory testing and *in*-*situ* evaluation. The key specifications of the temperature sensors used in this study are summarized in Table 1. The IRR sensor is designed to operate in environments having 0% to 100% RH. But, it is not proclaimed to be applicable in wastewater industry for measuring surface temperature. The IRR sensor is placed inside the enclosure with an angle of inclination 45° focusing on the surface of interest. An optical window is placed in front of the IRR sensor. One of the biggest challenge to monitor the surface temperature variations through non-contact surface temperature measurements is the high humidity levels in sewer air. In order to mitigate the effects of higher relative humidity conditions, we utilized a custom-made optical window to suit the sewer application. The optical window used in this study is made up of mono-crystalline germanium with wafer structure having 0.5 mm thickness, diameter of 26 mm and size tolerance of ±0.1 mm. In order to prevent the surface fogging, the optical window has undergone nanometer coating using the fluorine and silicon group. This optical window reduces the effects of water bands or atmospheric interference below 8 *μ*m and above 14 *μ*m. Besides the temperature sensors, the sensing unit accommodates electrical resistivity based moisture sensor within the sensor enclosure.

**Table 1:** Specifications of the temperature sensors used in this study.

Name	IRR Sensor (SI-111)	Thermistor Sensor (THERM-EP)
Measurement Range	−30 °C to +65 °C	−40 °C to +80 °C
Operating Temperature Range	−55 °C to +80 °C	−40 °C to +80 °C
Accuracy	±0.2 °C (−10 °C to +65 °C)	±0.05 °C
Resolution	0.01 °C	0.01 °C

**Figure 7:**
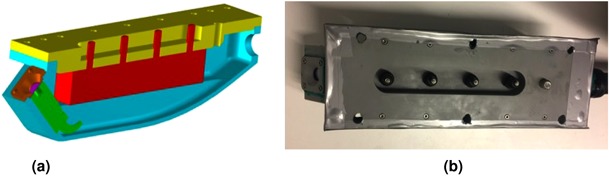
Sensing unit. (**a**) 3D model of the sensor enclosure showing the placement of sensors. (**b**) Picture of the sensor enclosure prior to sewer testing.

Figure 7a presents the 3D model of the sensor enclosure showing the placement of sensors within the package, where the green color indicates the IRR sensor and the red color indicates the moisture sensor. All the sensors were packaged in a custom designed PVC enclosure to suit the aggressive sewer environment. The enclosure was fabricated through Computer Numerical Control (CNC) milling process from the block of PVC material. The fasteners used in the sensor enclosure are produced from A4 grade 316 stainless steel material, which affords a higher level of resistance to corrosive environments. Then, to prevent moisture ingress into the sensor enclosure, electrical sealing compound (Henley’s Green Compound) of insulating type was applied between the enclosure body and sensor lid. Figure 7b shows the developed sensing unit prior to field testing, where the sensors were enclosed within it. The sensor enclosure was designed to accommodate easy carry and easy mount in sewers. 4-core cable with a drain wire was used with the IRR sensor and 2-Core cable was used with the thermistor sensor to extend 20 meters long for obtaining measurements. The cables carrying sensor measurement was enclosed in an IP65 rated PVCu spiral coated flexible conduit with an outer diameter of 27.5 mm and an inner diameter of 22 mm. This cable is suitable for operating temperature range from −5 °C to +60 °C. The joints connecting the sensor enclosure and the conduit were sealed with the thread sealing cord. In addition, the electrical sealing compound was applied to provide the moisture barrier. An IP66 rated enclosure made up of Acrylonitrile Butadiene Styrene (ABS) material was used to enclose the data loggers.

### Laboratory study

In the laboratory investigations, experiments to study the performance of optical window, effects of incident angle, limit of detection, distance, lighting condition, reproducibility, humidity conditions, varying surface temperature conditions were conducted. A humidity-controlled chamber was designed and developed in the laboratory for evaluating the IRR sensor in high humidity conditions. This chamber utilizes ultrasound humidification technology based air humidifier (LB 44, Beurer) for humidification inside the chamber and humidity sensor (DHT22, Aosong Electronics Co., Ltd) having 2–5% accuracy is used to measure the chamber’s RH. For assessing the germanium optical window coated with an antifog material, the sensing unit was placed inside the chamber and set to different RH levels such as 80% RH, 90% RH and 100% RH. A visual assessment of fogging on the lens was performed. For the statistical performance evaluation of laboratory experimentation, MAPE and RMSE were used as metrics, where *T*_*RIT*_ is the reference surface temperature measure from the thermistor sensor and *T*_*IRR*_ is the surface temperature measure from the IRR sensor, *n* is the number of samples and *t* is the instantaneous time. The MAPE and RMSE used in the laboratory experimentations are given below in Eqs 1 and 2 respectively.

1 $$MAPE=\frac{\mathrm{100 \% }}{n}\,\sum _{t=1}^{n}\,|\frac{{T}_{RIT}-{T}_{IRR}}{{T}_{RIT}}|$$

2 $$RMSE={[\frac{1}{n}\sum _{t=1}^{n}{({T}_{RIT,t}-{T}_{IRR,t})}^{2}]}^{1/2}$$

While evaluating the effects of incident angle, the IRR sensor was positioned at different angles so that the sensor focuses the concrete surface of interest proximity to reference thermistor. The measurements from the sensors were collected and relative RMSE was computed for comparing the IRR sensor performance at different incident angles. This experiment was conducted in the laboratory conditions having the room temperature of 23 °C and dark lighting condition. Also, the sensor has 22° half angle field of view, which makes the sensor to have the limit of detection in focusing the target surface.

The IRR sensor was kept at different distances such as 10 cm, 20 cm, 30 cm, 40 cm and 50 cm from the target surface. This experiment was conducted by placing the sensor at a 90° angle to the concrete surface in both illuminated and dark lighting conditions. Then, MAPE was used as a metric for performance evaluation to study the effects of distance and lighting condition during measurements. Further, the reproducibility of the measurement by the IRR sensor was accessed by taking repetitive measurements. These experiments were conducted under same operating conditions such as having the same incident angle, distance and lighting condition. From the reproducibility analysis experiments, the IRR sensor produced MAPE less than 2.5%.

In order to understand the effects of humidity during the IRR sensor measurements, the IRR sensor was exposed to different humidity conditions such as 80% RH, 90% RH and 100% RH inside the humidity chamber focusing the sewer exposed concrete. Then, the IRR sensor readings and the reference sensor readings were used to compute RMSE and MAPE for analyzing the humidity effects during measurements.

For comprehending the IRR sensing performance at varying surface temperatures, we placed the IRR sensor inside the humidity chamber and set it to 100% RH. A heating pad (HP-150, Auber Instruments) was also kept inside the chamber to increase the surface temperature levels to 25 °C, 30 °C, and 35 °C. The reference thermistor sensor was affixed on the pad and IRR sensor was positioned to focus it. Then, RMSE and MAPE were used as metrics to analyze the effects of varying surface temperature.

### Field application

To demonstrate the performance of the IRR sensor in the sewer conditions, the sensing unit was installed near the crown of the concrete sewer pipe. Sewer data from the sensors were transmitted to the monitoring unit constructed outside the sewer pipe through cables. No direct access was provided to the sensing unit during the field testing. The sensors were controlled and the data from the sensors were accessed from the monitoring station. The sensor suite was powered by using 12 V 100Ah rechargeable heavy duty batteries. In this field application, the sewer suite did not have an infrastructure to communicate remotely. Therefore, an operator checked the condition of the sensor suite once in a week and swapped fully charged batteries throughout the field trial. Besides the sensors inside the sensing unit, two free-standing thermistor sensor was used to measure the sewer air temperature and ambient temperature of the field location. The data-logger (TSM-1, ICT International) having five differential ended analogue channels were used for logging all the sensor measurements. The field monitoring campaign was carried out for about 96 days during the summer period in the Sydney city of Australia between 03^rd^ November 2016 and 07^th^ February 2017. The field application was done in a sewer having H_2_S levels approximately ranging from 2–5 ppm.

### Surface temperature correction

The ratio of radiant energy emitted by the surface to that emitted by a blackbody at the same temperature is known as emissivity^34^. The estimated emissivity of the surface is calculated based on the mathematical expression^34^ shown in Eq. 3.

3 $${\varepsilon }_{T}={\varepsilon }_{IR}{[\frac{{T}_{IRR}}{{T}_{RIT}}]}^{4}$$

where *ε*_*T*_ is the estimated emissivity of the surface, *ε*_*IR*_ is the set emissivity of the IRR sensor, T_*IRR*_ is the surface temperature measured by the IRR sensor and T_*RIT*_ is the surface temperature measured by the reference instrument thermistor. In this study, *ε*_*T*_ is determined by using the sensor data of about 5 days. The mean value of *ε*_*T*_ is denoted by *μ*. The value of *ε*_*IR*_ for the IRR sensor is 0.98. The surface temperature measurements from the IRR sensor is corrected by using the Eq. 4, where the Corrected T_*IRR*_ is the corrected surface temperature measure of the IRR sensor measurements. Also, the *ε*_*T*_ in the below equation denotes *μ*(*ε*_*T*_).

4 $$Corrected\,{T}_{IRR}={[\frac{{\varepsilon }_{T}}{{\varepsilon }_{IR}}]}^{\mathrm{1/4}}{T}_{IRR}$$

The RMSE was used as a statistical performance metric for evaluating the temperature correction after determining the emissivity coefficient.

### Post-deployment validations

After completing the field trial campaign, the sensor system was brought to the laboratory for post-deployment validations. The experimentation was performed in the dark lighting conditions of the laboratory similar to the confined sewer system. The IRR sensor was measuring the surface temperature by placing the sewer exposed optical window and a new optical window to pursue comparative analysis. The measurements were obtained from each sensor at certain time intervals and then, MAPE was used as a statistical metric to compute the relative percentage error between the IRR sensor and benchmark measures.

## Data Availability

In this study, the sensor data analyses and plots were achieved using the numerical computing software Matrix Laboratory (Matlab) version Matlab R2016b. The data and software code is available upon request from the corresponding author.
